# A tripe diffusion bioconvective model for thixotropic nanofluid with applications of induced magnetic field

**DOI:** 10.1038/s41598-024-58195-4

**Published:** 2024-04-08

**Authors:** Mohammed A. Albedah, Zhixiong Li, Iskander Tlili

**Affiliations:** 1 Department of Physics, College of Sciences, Al-Zulfi, Majmaah University, 11952 Al-Majmaah, Saudi Arabia; 2 Faculty of Mechanical Engineering, Opole University of Technology, 45-758 Opole, Poland; 3 Yonsei Frontier Lab, Yonsei University, Seoul, 03722 South Korea

**Keywords:** Thixotropic nanofluid, Bioconvective flow, Triple diffusion phenomenon, Stagnation point flow, Numerical computations, Biomaterials, Nanoscale materials, Applied mathematics

## Abstract

Owing to enhanced thermal characteristics of nanomaterials, multidisciplinary applications of such particles have been utilized in the industrial and engineering processes, chemical systems, solar energy, extrusion processes, nuclear systems etc. The aim of current work is to suggests the thermal performances of thixotropic nanofluid with interaction of magnetic force. The suspension of microorganisms in thixotropic nanofluid is assumed. The investigation is further supported with the triple diffusion flow. The motivations for considering the triple diffusion phenomenon are associated to attaining more thermal applications. The flow pattern is subject to novel stagnation point flow. The convective thermal constraints are incorporated. The modeled problem is numerically evaluated by using shooting technique. Different consequences of physical parameters involving the problem are graphically attributed. The insight analysis is presented for proposed problem with different engineering applications. It is claimed that induced magnetic field enhanced due to magnetic parameter while declining results are observed for thixotropic parameter. The heat transfer enhances due to variation of Dufour number. Furthermore, low profile of nanoparticles concentration has been observed for thixotropic parameter and nano-Lewis number.

## Introduction

A significant research is presented for nanofluids by scientists in current century. The main motivation of nanomaterials is due to extra peak thermal properties which make these particles more dynamic. The nanofluids are suspension of nanoparticles like oxides and carbides in base liquids. Based on excellent thermal performances, promising applications of nanomaterials have been predicted in the thermal management processes and industrial systems. The prime role of nanofluid can also be justified in the solar energy, different chemical reaction, heat transfer systems, heat exchangers etc. Moreover, in the biomedical area, the use of nanomaterials is suggested for targeted the drug delivery, destroying the suspicious cells, chemo-theory etc. Owing to stable and versatility heat transfer capacitance, the nanomaterials are concluded as a best energy source and thermal performances. Various attempts are reported for inspect the judgement of boosted thermal performances. For instance, Nguyen et al.^1^ explored the visualization of boosted thermal via utilizing the nanoparticles with square heat source. Ikram et al.^2^ examined the parallel plates flow with utilizing the metallic particles with help of novel fraction. Gowda et al.^3^ reported the ferromagnetic analysis for heat transfer phenomenon subject to entertain the features of magnetic dipole. Li et al.^4^ performed the optimized outcomes for nanofluids flow in porous media frame. Turki et al.^5^ discussed the unique performances of nano-materials to Access the applications in the electronics systems. AlBaidani et al.^6^ analyzed the ternary hybrid nanofluid heating determination for nanofluid by discussing the external shape features. The magnetic dioxide nanoparticles flow interacting against the car radiator as reported by Kumar et al.^7^. Tian et al.^8^ investigated the fractional impact of carbon nanotubes with dispersion of metallic particles. Ghachem et al.^9^ focused the externally supported heat transfer flow of nanoparticles with external heat source. Yasir et al.^10^ commuted the numerical assessment of nanoparticles problem with irregular heat conduction flow. Rehman et al.^11^ focused to Sutterby nanofluid with radiative impact. Mohapatra et al.^12^ analyzing the Lorenz force impact for copper–water based nanofluids by presenting a micro-convection model. Study of hybrid nanofluid with supported features of radiation was claimed by Ahmad et al.^13^. Darvesh et al.^14^ noticed the cross nanofluid aspects in heat transfer problem due to infinite shear rate. Obalalu et al.^15^ evaluated the two phase nanofluid properties in entropy generated flow accounted by vertical plate. The 3D flow of nanofluid with fluctuated viscosity role was inspected by Darvesh et al.^16^. Ige et al.^17^ executed the transient regime flow of blood liquid with interaction of hybrid nanofluid. Darvesh et al.^18^ expressed the variable chemical reactive flow due to cross nanofluid with significance of control of global warming. Maatoug et al.^19^ proceeded the zero mass thermal impact for spiraling disk flow by using the tangent hyperbolic fluid. Li et al.^20^ executed the fundamentals of heat transfer with copper nanoparticles with complex channel. The associated of magnetic dipole due to shear viscosity flow was evaluated by Darvesh et al.^21^. Ali et al.^22^ extended the heat transfer analysis for ferromagnetic nanoparticles with viscous dissipation. Hussain et al.^23^ analyzed the 3D nanofluid flow with heat sink applications. The convective heat transfer applications associated to cross nanofluid was observed by Ali et al.^24^.

Bioconvection phenomenon is based on the macroscopic inspection of fluids following the diverse density associated to the collective transport of microorganisms. The swimming of microorganisms is usually self-oriented in the upper regime of fluid which makes it relatively denser. Such diverse situation in the microorganisms creates the instability which effected the flow pattern in focused system. Key importance is depicted for bioconvection phenomenon with diligent applications in the biotechnology and environmental systems. In bio-fuels, fertilizers, bio-sensors and enzymes, the bioconvection convection applications are easily defended. The bioconvection assessment is further supported with the fascinating applications in the oil recovery, soil sciences and petroleum engineering. The swimming of microorganisms with suspension of nanomaterials is important to improve the stability impact. Koriko et al.^25^ defined the bioconvection aspects for thixotropic nanofluid endorsing the vertical space. Henda et al.^26^ performed the bioconvective analysis for magnetized third grade nanomaterial along with interpreting outcomes of activation energy. Tong et al.^27^ discussed the slip effects for bioconvective flow and modeled the problem with help of updated Fourier approach. Ullah et al.^28^ reported the 3D flow of nanofluid with decomposed aspects of microorganisms under absorption effects. The couple stress bioconvective analysis under the significant claim of biofuels was predicted by Khan et al.^29^. Ahmed et al.^30^ analyzed the tangent hyperbolic nanofluid with microorganisms. Alharbi et al.^31^ pronounced the micropolar bioconvective analysis for double radiative flow. Tabrez et al.^32^ analyzed the bioconvective analysis for Sutterby nanofluid due to heat surface.

The induced magnetic field is associated to the induction of magnetic force produced due to substance due to fluctuation of electric force. This phenomenon of induced magnetic field is key concept in the electromagnetism. This phenomenon is based on Faraday’s theory of electromagnetic induction. The induced magnetic force conveys important role of the electrical engineering, electronics, physical systems and electromagnetic theory Hayat et al.^33^ reported the induction of magnetic force for viscoelastic fluid. Gosh et al.^34^ discussed the magenta force performances for double diffusion flow associated to bioconvective Casson flow. Shatnawi et al.^35^ analyzed the induced magnetic force analysis against the uniformly dispersed hybrid nanoparticles. Shilpa and Leela^36^ discussed the interaction of induced magnetic force for annuls flow of Oldroyd-B nanofluid. Khan et al.^37^ examined the similar features for water-based hybrid nanofluid. Gogoi et al.^38^ announced the two- layer thin film flow subject to induced magnetic field.

In above stated literature, it is clear that that there have been different investigations on the applications of nanofluids. However, less attention has been paid towards the significance of induced magnetic force associated to the triple diffusion nanofluid flow. In order to fulfil this research gape, the objective of this analysis is to analyze the significance of triple diffusion flow of thixotropic nanofluid in presence of induced magnetic force features. The flow pattern is followed by stagnation point phenomenon. Following are the main objective of current research:

- Develop a mathematical model for triple diffusion flow of thixotropic nanofluid with suspension of microorganisms.
- Predicts the induction of magnetic force associated to the thixotropic nanofluid flow.
- Evaluates the fluctuation of thermal problem in presence of radiation effects.
- Examines the role of convective boundary constraints for heat and fluid flow problem.
- Perform the numerical computations by using shooting technique for modeled nonlinear problem.
- Analyze the characteristics of temperature profile, solutal concentration, nanoparticles concentration and microorganisms with variation of involved parameters.

It is worth mentioning that in all available nanofluid studies, no such effects are predicted. A detailed physical impact of problem has been presented.

## Statement of the problem

**Figure 1:**
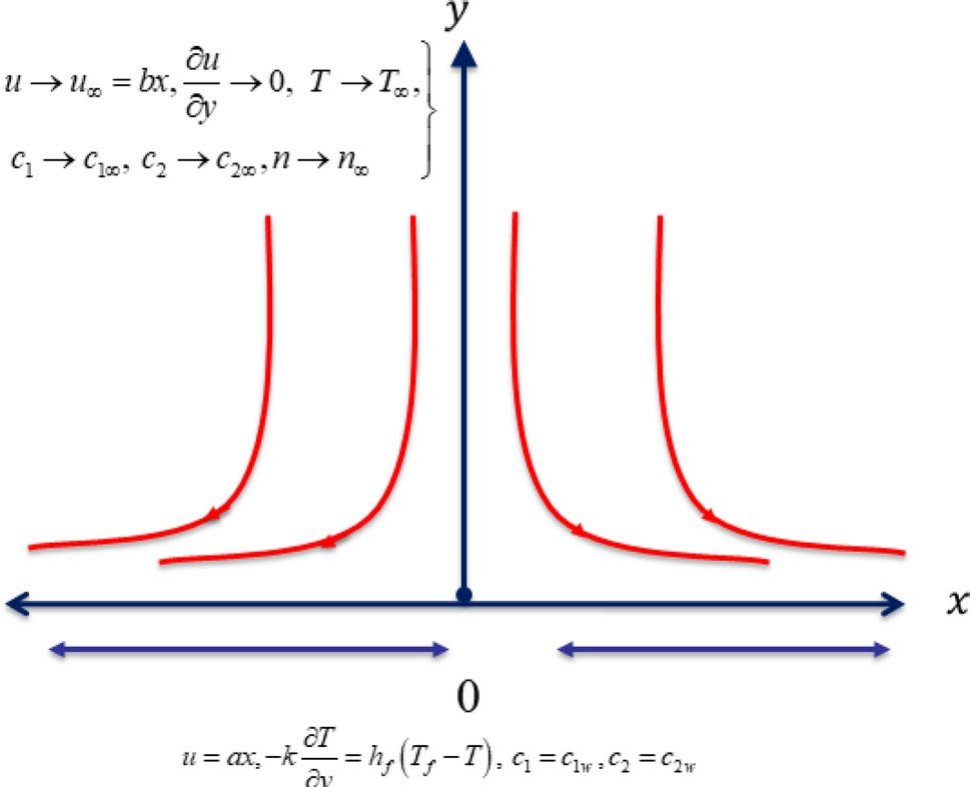
Flow configuration of the problem.

Let us develop a mathematical model for bioconvective flow of thixotropic nanofluid with applications of induced magnetic force. The stretching surface with uniform velocity attains the flow. The flow configuration for current model is presented in Fig. 1. The model is developed under following flow constraints:

- A two-dimensional (2D) stagnation point flow of thixotropic nanofluid due to moving surface is assumed.
- The strong magnetic force is interpreted with applications of magnetic induction.
- For stagnation point flow, the moving surface velocities are expressed are $u = ax$ and $u = bx.$
- The velocity component $u$ is taken along horizontal direction while illustration of velocity component is defined via $v$.
- The horizontal induced magnetic force component is expressed with $H_{1}$ while $H_{2}$ is along normal way.
- The radiative phenomenon is utilized in energy equation.

Following to constraints, the modeled problem is governed by following system of equations^25,33^:

1 $$\frac{\partial u}{{\partial x}} + \frac{\partial v}{{\partial y}} = 0,$$

2 $$\frac{{\partial H_{1} }}{\partial x} + \frac{{\partial H_{2} }}{\partial y} = 0,$$

3 $$\begin{gathered} u\frac{\partial u}{{\partial x}} + v\frac{\partial u}{{\partial y}} = \nu \frac{{\partial^{2} u}}{{\partial y^{2} }} - \frac{{6\omega_{1} }}{{\rho_{f} }}\left( {\frac{\partial u}{{\partial y}}} \right)^{2} \frac{{\partial^{2} u}}{{\partial y^{2} }} + \frac{{4\omega_{2} }}{{\rho_{f} }}\left[ {\frac{\partial u}{{\partial y}}\frac{{\partial^{2} u}}{{\partial y^{2} }}\left( {u\frac{{\partial^{2} u}}{\partial x\partial y} + v\frac{{\partial^{2} u}}{{\partial y^{2} }}} \right) + \left( {\frac{\partial u}{{\partial y}}} \right)^{2} \left( \begin{gathered} u\frac{{\partial^{3} u}}{{\partial x\partial y^{2} }} + \frac{{\partial^{3} u}}{{\partial y^{3} }} \hfill \\ + \frac{\partial u}{{\partial y}}\frac{{\partial^{2} u}}{\partial x\partial y} \hfill \\ + \frac{\partial v}{{\partial y}}\frac{{\partial^{2} u}}{{\partial y^{2} }} \hfill \\ \end{gathered} \right)} \right] \hfill \\ + u_{\infty } \frac{{\partial u_{\infty } }}{\partial x} + \frac{{\sigma B_{0}^{2} }}{{\rho_{f} }}\left( {u_{\infty } - u} \right) - \frac{\mu }{{4\pi \rho_{f} }}H_{e} \frac{{\partial H_{e} }}{\partial x} + \frac{\mu }{{4\pi \rho_{f} }}\left( {H_{1} \frac{{\partial H_{1} }}{\partial x} + H_{2} \frac{{\partial H_{1} }}{\partial y}} \right), \hfill \\ \end{gathered}$$

4 $$u\frac{{\partial H_{1} }}{\partial x} + v\frac{{\partial H_{2} }}{\partial y} = H_{1} \frac{\partial u}{{\partial x}} + H_{2} \frac{\partial u}{{\partial y}} + u_{e} \frac{{\partial^{2} H_{1} }}{{\partial x^{2} }},$$

5 $$\begin{gathered} u\frac{\partial T}{{\partial x}} + v\frac{\partial T}{{\partial y}} = \left( {\gamma_{m} + \frac{{16\sigma_{1} T_{\infty }^{3} }}{{3\left( {\rho c} \right)_{p} \kappa_{1} }}} \right)\frac{{\partial^{2} T}}{{\partial y^{2} }} + \Lambda_{s} \left[ {D_{T} \frac{{\partial c_{1} }}{\partial y}\frac{\partial T}{{\partial y}} + \frac{{D_{T} }}{{T_{\infty } }}\left( {\frac{\partial T}{{\partial y}}} \right)^{2} } \right] \hfill \\ + DK_{TC} \left( {\frac{{\partial^{2} c_{1} }}{{\partial y^{2} }}} \right), \hfill \\ \end{gathered}$$

6 $$u\frac{{\partial c_{1} }}{\partial x} + v\frac{{\partial c_{1} }}{\partial y} = D_{s} \frac{{\partial^{2} c_{1} }}{{\partial y^{2} }} + DK_{CT} \frac{{\partial^{2} T}}{{\partial y^{2} }},$$

7 $$u\frac{{\partial c_{2} }}{\partial x} + v\frac{{\partial c_{2} }}{\partial y} = D_{B} \frac{{\partial^{2} c_{2} }}{{\partial y^{2} }} + \frac{{D_{T} }}{{T_{\infty } }}\frac{{\partial^{2} T}}{{\partial y^{2} }},$$

8 $$u\frac{\partial n}{{\partial x}} + v\frac{\partial n}{{\partial y}} + \frac{{b_{1} w_{1} }}{{\left( {C_{w} - C_{\infty } } \right)}}\left[ {\frac{\partial }{\partial y}\left( {n\frac{{\partial c_{1} }}{\partial y}} \right)} \right] = D_{m} \left( {\frac{{\partial^{2} n}}{{\partial y^{2} }}} \right),$$

with material constants $\left( {\omega_{1} ,\omega_{2} } \right),$ fluid density $\left( {\rho_{f} } \right),$ kinematic viscosity $\nu$, free stream velocity $\left( {u_{\infty } } \right)$, $x -$ magnetic field edge $\left( {H_{e} } \right),$ dynamic viscosity $\left( \mu \right)$, temperature $T$, Soret diffusivity $\left( {DK_{TC} } \right)$, heat capacity nanofluid ratio $\left( {\Lambda_{s} } \right)$, thermophoretic coefficient $\left( {D_{T} } \right)$, Brownian diffusion $\left( {D_{B} } \right)$, solutal concentration $\left( {c_{1} } \right)$, nanoparticle volume fraction $\left( {c_{2} } \right)$, microorganisms density $\left( n \right)$, swimming cells speed $\left( {w_{1} } \right)$ and chemotaxis constant $\left( {b_{1} } \right).$

Soret diffusivity $\left( {DK_{TC} } \right)$ and Soret diffusivity $\left( {DK_{CT} } \right)$.

The flow assumptions are^25^:

9 $$\left. {u = ax,v = 0,\frac{{\partial^{2} u}}{{\partial y^{2} }} = 0,\frac{{\partial H_{1} }}{\partial y} = H_{2} = 0, - k\frac{\partial T}{{\partial y}} = h_{f} \left( {T_{f} - T} \right),c_{1} = c_{1w} ,c_{2} = c_{2w} ,\,n = n_{w} \,at\,y = 0,} \right\}$$

10 $$u \to u_{\infty } = bx,\frac{\partial u}{{\partial y}} \to 0,\,\,\,H_{1} = H_{e} \left( x \right) \to H_{0} \left( x \right),T \to T_{\infty } ,\,c_{1} \to c_{1\infty } ,\,\,c_{2} \to c_{2\infty } ,n \to n_{\infty } \,\,as\,\,y \to \infty .$$

where $a$ and $b$ are stretching constants, $k$ is thermal conductivity and $h_{f}$ heat transfer coefficient.

New variables are^25^:

11 $$\left. \begin{gathered} v = - \sqrt {a\nu } f\left( \eta \right),H_{2} = - H_{0} \sqrt {\frac{\nu }{a}} l\eta ,\eta = \sqrt {\frac{a}{\nu }} y,u = axf^{\prime}\left( \eta \right), \hfill \\ \psi \left( \eta \right) = \frac{{c_{1} - c_{1\infty } }}{{c_{1w} - c_{1\infty } }},\phi \left( \eta \right) = \frac{{c_{2} - c_{2\infty } }}{{c_{2w} - c_{2\infty } }},\theta \left( \eta \right) = \frac{{T - T_{\infty } }}{{T_{f} - T_{\infty } }},\chi \left( \eta \right) = \frac{{n - n_{\infty } }}{{n_{w} - n_{\infty } }}. \hfill \\ \end{gathered} \right\}.$$

The formulated system is:

12 $$f^{\prime\prime\prime} - f^{\prime 2} + ff^{\prime\prime} + A^{2} + M\left( {l^{\prime 2} - ll^{\prime\prime} - 1} \right) + \beta_{1} \left( {f^{\prime\prime}} \right)^{2} f^{\prime\prime\prime} + \beta_{2} \left( \begin{gathered} f^{\prime}\left( {f^{\prime\prime}} \right)^{2} f^{\prime\prime\prime} + \left( {f^{\prime\prime}} \right)^{4} \hfill \\ - ff^{\prime\prime}\left( {f^{\prime\prime}} \right)^{2} - f\left( {f^{\prime\prime}} \right)^{2} f^{\prime \prime \prime \prime } \hfill \\ \end{gathered} \right),$$

13 $$\delta l^{\prime\prime\prime} - lf^{\prime\prime} + fl^{\prime\prime} = 0,$$

$$\left( {1 + \frac{4}{3}Rd} \right)\theta ^{\prime\prime} + \Pr \left[ {f\varphi^{\prime} + Nb\theta^{\prime}\varphi^{\prime} + Nt\left( {\theta^{\prime}} \right)^{2} + \left( {Nd} \right)\varphi^{\prime\prime}} \right] = 0,$$

14 $$\psi^{\prime\prime} + \left( {Le} \right)f\psi^{\prime} + Ld\theta^{\prime\prime} = 0,$$

15 $$\phi^{\prime\prime} + Ln\left( {f\phi^{\prime}} \right) + \frac{Nt}{{Nb}}\theta^{\prime\prime} = 0,$$

16 $$\chi^{\prime\prime} + Lb\chi^{\prime} - Pe\left[ {\varphi^{\prime}\left( {\chi + \sigma } \right) + \chi^{\prime}\varphi^{\prime}} \right] = 0,$$

with boundary conditions:

17 $$\left. \begin{gathered} f\left( 0 \right) = 0,f^{\prime}\left( 0 \right) = 1,f^{\prime\prime\prime}\left( 0 \right) = 0,l\left( 0 \right) = l^{\prime\prime}\left( 0 \right) = 0, \hfill \\ \theta ^{\prime}\left( 0 \right) = - Bi\left[ {1 - \theta \left( 0 \right)} \right],\psi \left( 0 \right) = 1,\phi \left( 0 \right) = 1,\chi \left( 0 \right) = 1, \hfill \\ \end{gathered} \right\}$$

18 $$\left. \begin{gathered} f^{\prime}\left( \infty \right) \to A,\,\,\,\,f^{\prime\prime}\left( \infty \right) \to 0,\,\,\,\,\,l^{\prime}\left( \infty \right) \to 1,\,\, \hfill \\ \theta \left( \infty \right) \to 0,\psi \left( \infty \right) \to 0,\phi \left( \infty \right) \to 0,\chi \left( \infty \right) \to 0. \hfill \\ \end{gathered} \right\}$$

where $A = \frac{b}{a}$ is velocity ratio constant, thixotropic parameters $\beta_{1} = - 6\omega_{1} a^{3} x^{2} /\rho_{f} \nu^{2} ,\beta_{2} = 4\omega_{2} a^{4} x^{2} /\rho_{f} \nu^{2} ,$ reciprocal magnetic Prandtl number $\delta = \frac{{\mu_{e} }}{\nu },$ magnetic parameter $M = \frac{\mu }{{4\pi \rho_{f} }}\left( {\frac{{H_{0} }}{a}} \right)^{2} ,$ Brownian parameter $Nb = \Lambda_{s} D_{B} \left( {C_{w} - C_{\infty } } \right)/\nu$, thermophoresis constant $Nt = \Lambda_{s} D_{T} \left( {T_{w} - T_{\infty } } \right)/T_{\infty } \nu$, modified Dufour number $Nd = D_{TC} \left( {c_{1w} - c_{1\infty } } \right)/\gamma_{m} \left( {T_{w} - T_{\infty } } \right)$, regular Lewis number $Le = \nu /D_{s}$, Dufour Lewis coefficient $Ld = D_{CT} \left( {T_{w} - T_{\infty } } \right)/\gamma_{m} \left( {c_{1w} - c_{1\infty } } \right)$ and nano-Lewis coefficient $Ln = \nu /D_{B}$, radiation parameter $Rd = \frac{{16\sigma_{1} T_{\infty }^{3} }}{{3\kappa_{1} k}},$ thermal Biot number $Bi = \left( \frac{h}{k} \right)\sqrt {\frac{\nu }{a}}$, bioconvection Lewis number $Lb = \nu /D_{m}$ Peclet number $Pe = b_{1} w_{a} /D_{m} .$

Defining the relations for Nusselt number, Sherwood number and nano-Sherwood constant as:

19 $$Nu_{x} = \frac{{xq_{s} }}{{k\left( {T_{f} - T_{\infty } } \right)}},\,Sh_{x} = \frac{{xj_{s} }}{{D_{B} \left( {c_{1w} - c_{1\infty } } \right)}},Sh_{xn} = \frac{{xq_{m} }}{{D_{s} \left( {c_{2w} - c_{2\infty } } \right)}},Nn_{x} = \frac{{xj_{n} }}{{D_{n} \left( {n_{w} - n_{\infty } } \right)}}$$

where $q_{s}$ is the surface heat flux, $j_{s}$ expresses the surface solutal flux, $q_{m}$ be surface mass flux while $j_{n}$ denotes motile microorganisms flux which are defined as:

20 $$q_{s} = - k\left( {1 + \frac{{16\sigma_{1} T_{\infty }^{3} }}{{3\left( {\rho c} \right)_{p} \kappa_{1} }}} \right)\left( {\frac{\partial T}{{\partial y}}} \right)_{y = 0} ,j_{s} = - D_{s} \left( {\frac{{\partial c_{1} }}{\partial y}} \right)_{y = 0} ,q_{m} = - D_{B} \left( {\frac{{\partial c_{2} }}{\partial y}} \right)_{y = 0} ,j_{n} = - D_{m} \left( {\frac{\partial n}{{\partial y}}} \right)_{y = 0} ,$$

In view of dimensionless variables (Eq. 11), the dimensionless forms of above physical quantities are:

21 $$\left. \begin{gathered} Nu_{x} {\text{Re}}_{x}^{ - 0.5} = - \left( {1 + \frac{4}{3}Rd} \right)\theta^{\prime}\left( 0 \right), \hfill \\ Sh_{x} {\text{Re}}_{x}^{ - 0.5} = - \psi^{\prime}\left( 0 \right), \hfill \\ Sh_{n} {\text{Re}}_{x}^{ - 0.5} = - \phi^{\prime}\left( 0 \right), \hfill \\ Nn_{x} {\text{Re}}_{x}^{ - 0.5} = - \chi^{\prime}\left( 0 \right). \hfill \\ \end{gathered} \right\}$$

with ${\text{Re}}_{x} = u_{w} x/\nu$ (Reynolds constant).

## Numerical scheme

The formulated problem is resulted in terms of nonlinear differential equations for which exact solution cannot be examined directly. In order to evaluate the numerical solution of problem, shooting technique is implemented. The shooting technique is one of efficient solver to compute the approximate solution with convincing accuracy. For computations, MATLAB solver is used. The accuracy of numerical data is ensured and confirmed at 10^−7^. In order to start the simulations, the higher order system to first converted into first order approximations as follows:

22 $$\left. \begin{gathered} f = {\mathchar'26\mkern-10mu\lambda}_{1} , \, f^{\prime} = {\mathchar'26\mkern-10mu\lambda}_{2} , \, f^{\prime\prime} = {\mathchar'26\mkern-10mu\lambda}_{3} ,f^{\prime\prime\prime} = {\mathchar'26\mkern-10mu\lambda}_{4} ,f^{\prime \prime \prime \prime } = {\mathchar'26\mkern-10mu\lambda}_{4}^{\prime } , \hfill \\ l = {\mathchar'26\mkern-10mu\lambda}_{5} ,l^{\prime} = {\mathchar'26\mkern-10mu\lambda}_{6} ,l^{\prime\prime} = {\mathchar'26\mkern-10mu\lambda}_{7} ,l^{\prime\prime\prime} = {\mathchar'26\mkern-10mu\lambda}_{7}^{\prime } ,\theta = {\mathchar'26\mkern-10mu\lambda}_{8} , \, \theta^{\prime} = {\mathchar'26\mkern-10mu\lambda}_{9} ,\theta^{\prime\prime} = {\mathchar'26\mkern-10mu\lambda}_{9}^{\prime } , \hfill \\ \varphi = {\mathchar'26\mkern-10mu\lambda}_{10} , \, \varphi^{\prime} = {\mathchar'26\mkern-10mu\lambda}_{11} ,\varphi^{\prime\prime} = {\mathchar'26\mkern-10mu\lambda}_{11}^{\prime } ,\phi = {\mathchar'26\mkern-10mu\lambda}_{12} , \, \phi^{\prime} = {\mathchar'26\mkern-10mu\lambda}_{13} ,\phi^{\prime\prime} = {\mathchar'26\mkern-10mu\lambda}_{13}^{\prime } , \hfill \\ \chi = {\mathchar'26\mkern-10mu\lambda}_{14} ,\chi^{\prime} = {\mathchar'26\mkern-10mu\lambda}_{15} ,\chi^{\prime\prime} = {\mathchar'26\mkern-10mu\lambda}_{15}^{\prime } . \hfill \\ \end{gathered} \right\}$$

23 $${\mathchar'26\mkern-10mu\lambda}_{4}^{\prime } = \frac{{{\mathchar'26\mkern-10mu\lambda}_{4} - {\mathchar'26\mkern-10mu\lambda}_{4}^{2} + {\mathchar'26\mkern-10mu\lambda}_{1} {\mathchar'26\mkern-10mu\lambda}_{3} + A^{2} + M\left( {{\mathchar'26\mkern-10mu\lambda}_{6}^{2} - {\mathchar'26\mkern-10mu\lambda}_{5} {\mathchar'26\mkern-10mu\lambda}_{7} - 1} \right) + \beta_{1} \left( {{\mathchar'26\mkern-10mu\lambda}_{3} } \right)^{2} {\mathchar'26\mkern-10mu\lambda}_{4} + \beta_{2} \left( \begin{gathered} {\mathchar'26\mkern-10mu\lambda}_{2} \left( {{\mathchar'26\mkern-10mu\lambda}_{3} } \right)^{2} {\mathchar'26\mkern-10mu\lambda}_{4} + \left( {{\mathchar'26\mkern-10mu\lambda}_{3} } \right)^{4} \hfill \\ - {\mathchar'26\mkern-10mu\lambda}_{1} {\mathchar'26\mkern-10mu\lambda}_{3} \left( {{\mathchar'26\mkern-10mu\lambda}_{3} } \right)^{2} \hfill \\ \end{gathered} \right)}}{{\beta_{2} {\mathchar'26\mkern-10mu\lambda}_{1} \left( {{\mathchar'26\mkern-10mu\lambda}_{3} } \right)^{2} }},$$

24 $$\delta {\mathchar'26\mkern-10mu\lambda}_{7}^{\prime } = {\mathchar'26\mkern-10mu\lambda}_{5} {\mathchar'26\mkern-10mu\lambda}_{3} - {\mathchar'26\mkern-10mu\lambda}_{1} {\mathchar'26\mkern-10mu\lambda}_{7} ,$$

25 $${\mathchar'26\mkern-10mu\lambda}_{9}^{\prime } = \frac{{ - \left\{ {\Pr \left[ {{\mathchar'26\mkern-10mu\lambda}_{1} {\mathchar'26\mkern-10mu\lambda}_{11} + Nb{\mathchar'26\mkern-10mu\lambda}_{9} {\mathchar'26\mkern-10mu\lambda}_{11} + Nt\left( {{\mathchar'26\mkern-10mu\lambda}_{9} } \right)^{2} + \left( {Nd} \right){\mathchar'26\mkern-10mu\lambda}_{11}^{\prime } } \right]} \right\}}}{{1 + \frac{4}{3}Rd}},$$

26 $${\mathchar'26\mkern-10mu\lambda}_{11}^{\prime } = - Le\left( {{\mathchar'26\mkern-10mu\lambda}_{1} {\mathchar'26\mkern-10mu\lambda}_{11} } \right) - Ld{\mathchar'26\mkern-10mu\lambda}_{9}^{\prime } ,$$

27 $${\mathchar'26\mkern-10mu\lambda}_{13}^{\prime } = - Ln\left( {{\mathchar'26\mkern-10mu\lambda}_{1} {\mathchar'26\mkern-10mu\lambda}_{13}^{\prime } } \right) - \frac{Nt}{{Nb}}{\mathchar'26\mkern-10mu\lambda}_{9}^{\prime } ,$$

28 $${\mathchar'26\mkern-10mu\lambda}_{15}^{\prime } = - Lb{\mathchar'26\mkern-10mu\lambda}_{15} + Pe\left[ {{\mathchar'26\mkern-10mu\lambda}_{10} \left( {{\mathchar'26\mkern-10mu\lambda}_{14} + \sigma } \right) + {\mathchar'26\mkern-10mu\lambda}_{15} {\mathchar'26\mkern-10mu\lambda}_{11} } \right].$$

with

29 $$\left. \begin{gathered} {\mathchar'26\mkern-10mu\lambda}_{1} \left( 0 \right) = 0,{\mathchar'26\mkern-10mu\lambda}_{2} \left( 0 \right) = 1,{\mathchar'26\mkern-10mu\lambda}_{4} \left( 0 \right) = 0,{\mathchar'26\mkern-10mu\lambda}_{5} \left( 0 \right) = {\mathchar'26\mkern-10mu\lambda}_{7} \left( 0 \right) = 0, \hfill \\ {\mathchar'26\mkern-10mu\lambda}_{9} \left( 0 \right) = - Bi\left[ {1 - {\mathchar'26\mkern-10mu\lambda}_{8} \left( 0 \right)} \right],{\mathchar'26\mkern-10mu\lambda}_{10} \left( 0 \right) = 1,{\mathchar'26\mkern-10mu\lambda}_{12} \left( 0 \right) = 1,{\mathchar'26\mkern-10mu\lambda}_{14} \left( 0 \right) = 1, \hfill \\ \end{gathered} \right\}$$

30 $$\left. \begin{gathered} {\mathchar'26\mkern-10mu\lambda}_{2} \left( \infty \right) \to A,{\mathchar'26\mkern-10mu\lambda}_{3} \left( \infty \right) \to 0,{\mathchar'26\mkern-10mu\lambda}_{6} \left( \infty \right) \to 1, \hfill \\ {\mathchar'26\mkern-10mu\lambda}_{8} \left( \infty \right) \to 0,{\mathchar'26\mkern-10mu\lambda}_{10} \left( \infty \right) \to 0,{\mathchar'26\mkern-10mu\lambda}_{12} \left( \infty \right) \to 0,{\mathchar'26\mkern-10mu\lambda}_{14} \left( \infty \right) \to 0. \hfill \\ \end{gathered} \right\}$$

## Validation of results

The accuracy of solution is validated in Table 1 by comparing obtained solution with work of Mehmood and Iqbal^39^ and Ali et al.^40^. A fine agreement has been observed with these available studies (Table 2).

**Table 1:** Validation of results with available studies when $M = \beta_{1} = \beta_{2} = 0.$

$A$	Mehmood and Iqbal^39^	Ali et al.^40^	Present results
0.1	− 0.9694	− 0.9694	− 0.9695
0.2	− 0.9181	− 0.9181	− 0.9181
0.5	− 0.6673	− 0.6673	− 0.6673

**Table 2:** Numerical visualization for $- \theta^{\prime}\left( 0 \right), - \varphi^{\prime}\left( 0 \right), - \phi^{\prime}\left( 0 \right)$ and $- \chi^{\prime}\left( 0 \right)$ for flow parameters.

$\beta_{1}$	$\beta_{2} .$	$M$	$Nd$	$Nt$	$- \theta^{\prime}\left( 0 \right)$	$- \varphi^{\prime}\left( 0 \right)$	$- \phi^{\prime}\left( 0 \right)$	$- \chi^{\prime}\left( 0 \right)$
0.2	0.1	0.5	0.3	0.3	0.67532	0.62765	0.55455	0.48778
0.4					0.71325	0.63764	0.56655	0.49455
0.6					0.73626	0.65546	0.57424	0.51787
0.3	0.2				0.69426	0.64566	0.56033	0.50423
	0.6				0.73758	0.66013	0.57411	0.52978
	1.0				0.78326	0.67678	0.58568	0.52168
		0.4			0.71055	0.65657	0.60354	0.49443
		0.8			0.76465	0.67565	0.64157	0.53343
		1.2			0.79656	0.72345	0.67996	0.58677
			0.2		0.64626	0.57432	0.48556	0.51767
			0.4		0.62784	0.54676	0.45652	0.49768
			1.0		0.61564	0.52676	0.42376	0.46786
				0.2	0.67542	0.59546	0.54788	0.42022
				0.4	0.63064	0.57456	0.48462	0.41565
				0.6	0.57456	0.51452	0.45235	0.40654

## Results and discussion

**Figure 2:**
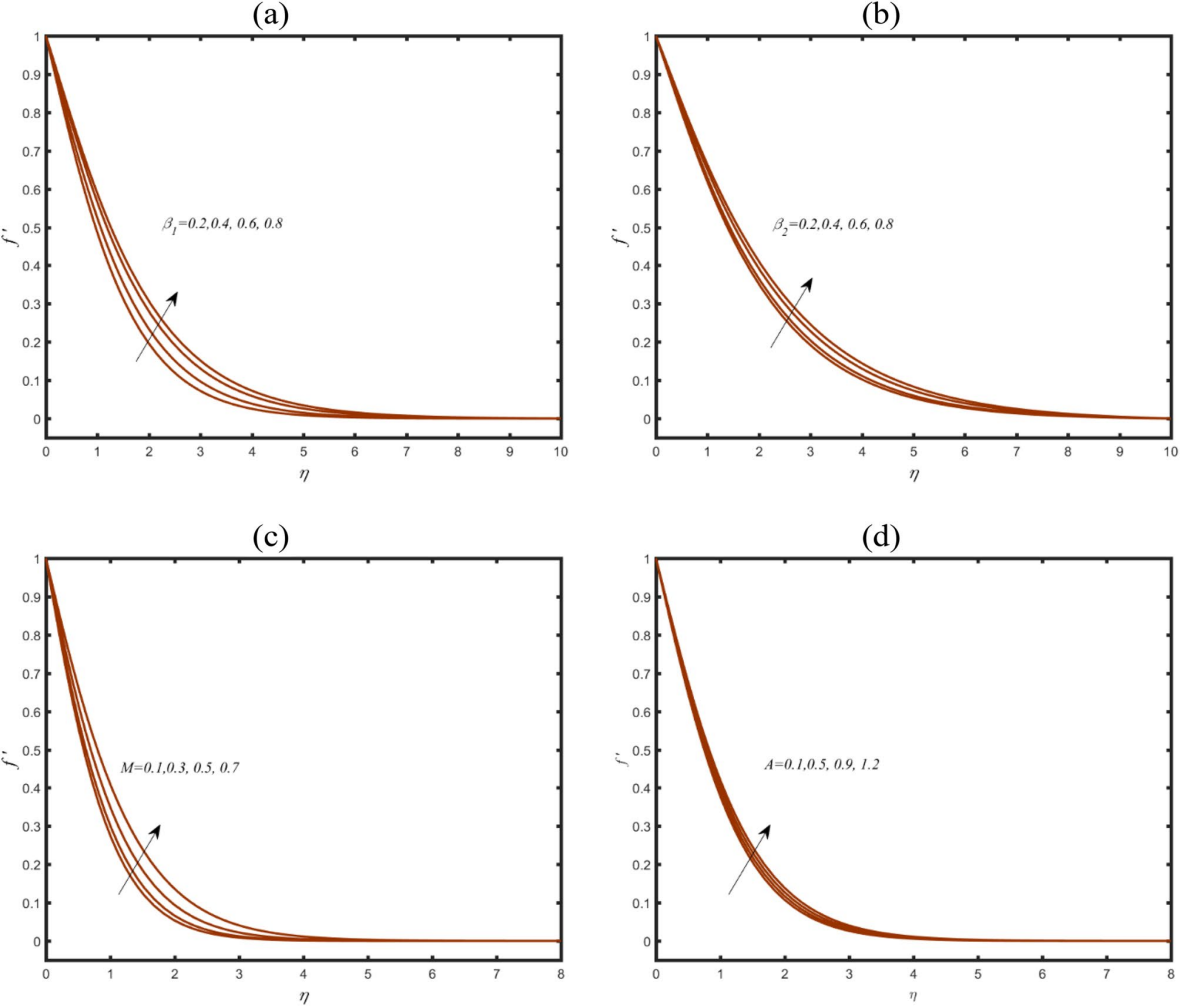
(**a**–**d**): Profile of $f^{\prime}$ due to (**a**) $\beta_{1}$ (**b**) $\beta_{2}$ (**c**) $M$ (**d**) $A$.

The modeled problem involves various parameters which present special role to inspect the bioconvective thermal phenomenon. Since stated problem is developed under the theoretical flow assumptions, so analysis is performed for some fixed range of involved parameters defined by $$0.2 \le \beta_{1} \le 0.8,$$ $$0.2 \le \beta_{2} \le 0.8,$$ $$0.1 \le M \le 0.7,$$ $$0.1 \le A \le 1.2,$$ $$0.1 \le \delta \le 0.8,$$ $$0.5 \le Nb \le 2.0,$$ $$0.2 \le Nd \le 0.8,$$ $$0.1 \le Le \le 1.2,$$ $$0.1 \le Ld \le 0.7,$$ $$0.1 \le Ln \le 0.7,$$ $$0.2 \le Nt \le 0.9,$$$$0.1 \le Pe \le 0.8$$ and $$0.2 \le Lb \le 1.2.$$ Figure 2a scrutinizes the insight of thixotropic parameters $$\beta_{1}$$ on velocity $$f^{\prime}$$. An upraise change in behavior of $$f^{\prime}$$ have been responded due to $$\beta_{1}$$. Physically, larger change in $$\beta_{1}$$ leads to less viscous effects associated to the shear force. Same increasing assessment in $$f^{\prime}$$ are predicted in Fig. 2b which covey the role of $$\beta_{2}$$. Physical judgement of such increment is associated to the exclusive rheological properties of thixotropic material. In this model, the shear thinning properties are fluctuated with different viscosity. For larger shear force, the viscosity declined which results an enhancement in velocity. Figure 2c preserving the visualization of role of magnetic parameter $$M$$ on $$f^{\prime}$$. The peak profile $$f^{\prime}$$ is appeared due to $$M$$. The induction of magnetic field strength the velocity profile. In Fig. 2d, the representation of $$f^{\prime}$$ is noticed against velocity ratio constant $$A$$. The fluid velocity is altered with enhancing trend subject to enlarging $$A$$. The velocity ratio constant presents the relative velocity of stretched surface to fluid flowing over it. This parameter justifies the role of stretched surface to effect the flow behavior.

**Figure 3:**
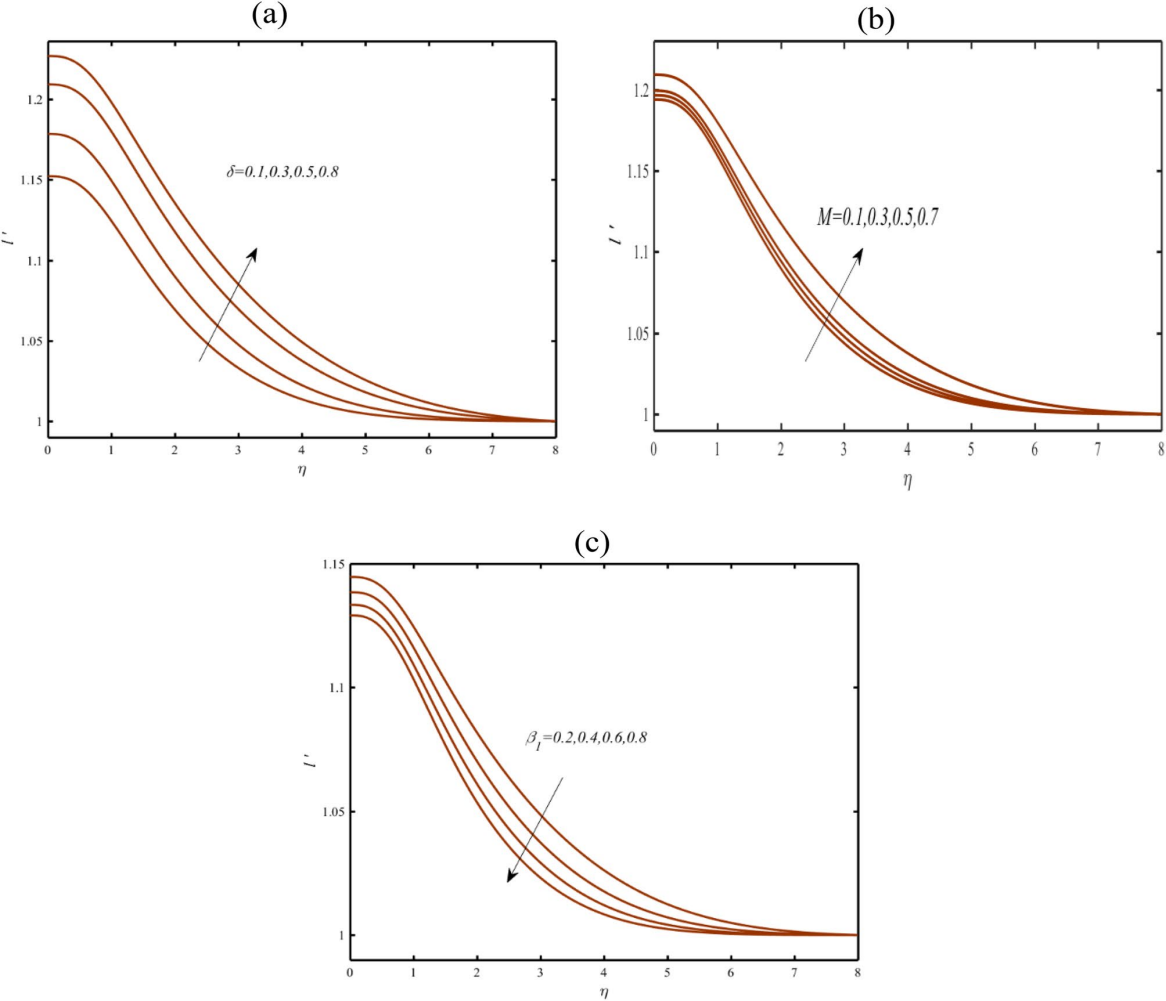
(**a**–**c**): Profile of $l^{\prime}$ due to (**a**) $\delta$ (**b**) $M$ (**c**) $\beta_{1}$.

Figure 3a determines the presentation of induced magnetic field profile $l^{\prime}$ due to reciprocal magnetic Prandtl number $\delta$. More strengthened prediction is visualized in $l^{\prime}$ against $\delta$. Physically, these increasing reflection is due to strong magnetic induction. Figure 3b presents the key observations for magnetic parameter $M$ for induced magnetic field profile $l^{\prime}$. The increasing change is depicted in $l$ for $M$. The insight analysis for $l^{\prime}$ with variation of thixotropic parameter $\beta_{1}$ is defended via Fig. 3c. The magnetic field profile become more enhancing due to $\beta_{1}$.

**Figure 4:**
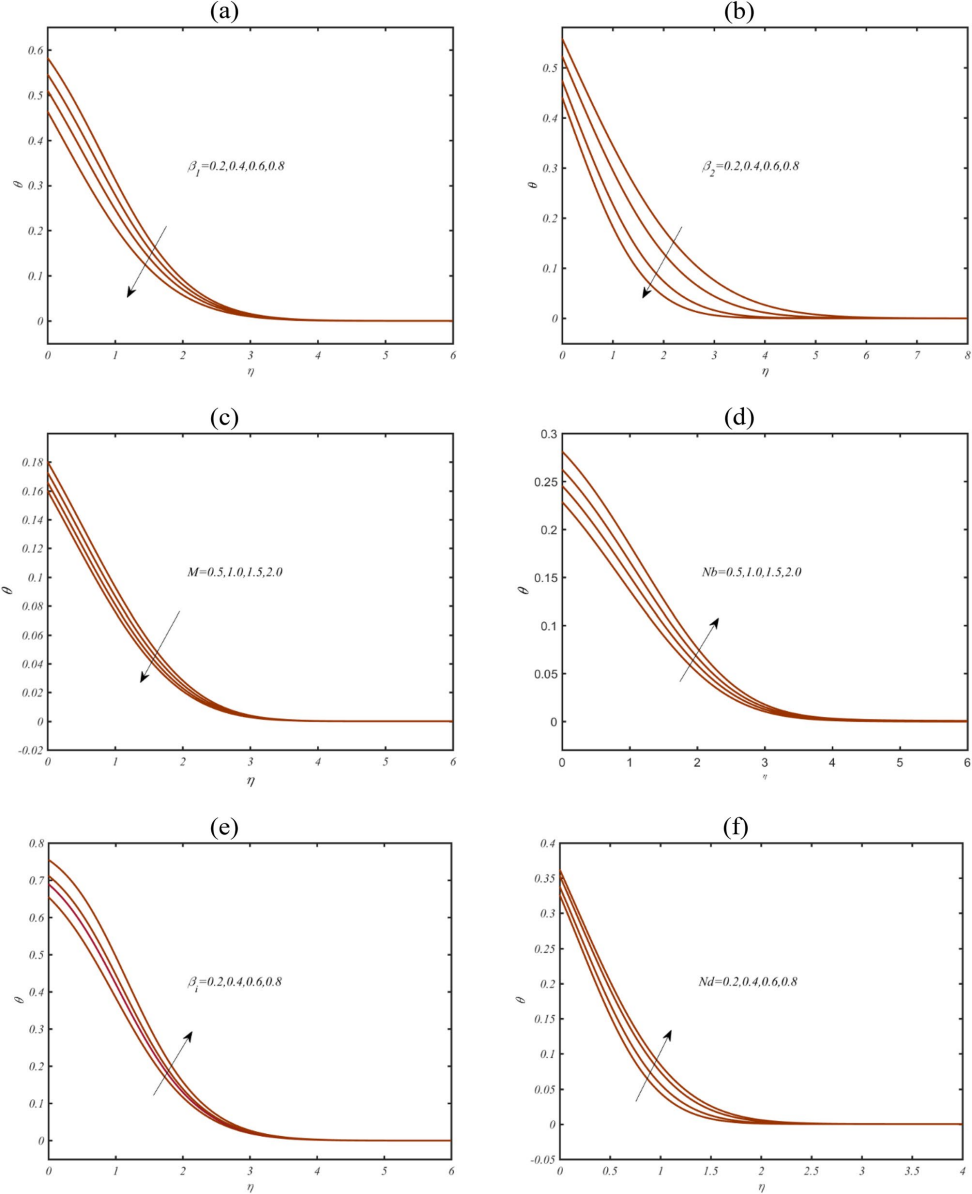
(**a**–**f**): Profile of $\theta$ due to (**a**) $\beta_{1}$ (**b**) $\beta_{2}$ (**c**) $M$ (**d**) $Nb$, (**e**)$Bi$ (**f**) $Nd$.

Figure 4a,b presents the investigation for temperature profile $\theta$ due to thixotropic parameters $\beta_{1}$ and $\beta_{2}$. A reduction has been evaluated for both thixotropic coefficients in profile of $\theta$. These declining change is due to rheological impact of non-Newtonian (thixotropic) model. Such novel rheological outcomes make this model more beneficiary in industrial processes. Figure 4c showing the features of magnetic parameter $M$ on $\theta$. The gradually reduction is exhibited in $\theta$ due to $M$. Figure 4d discloses the significance of Brownian parameter $Nb$ for visualization of $\theta$. The Brownian parameter is associated to the random movement of fluid particles which enriches the heat transfer rate. Figure 4e examined the profile of $\theta$ due to Biot number $Bi$. An enrich heat transfer is noted when role of $Bi$ is contributed. Physically, the Biot number presents the relation between coefficient of heat transfer. Figure 4f shows that for larger modified Dufour number $Nd$, $\theta$ enhances. Physically, the modified Dufour number represents the ratio between thermal diffusion to momentum diffusion. Larger values of $Nd$ leads to enhancement in thermal diffusion which corresponds to improvement in heat transfer.

**Figure 5:**
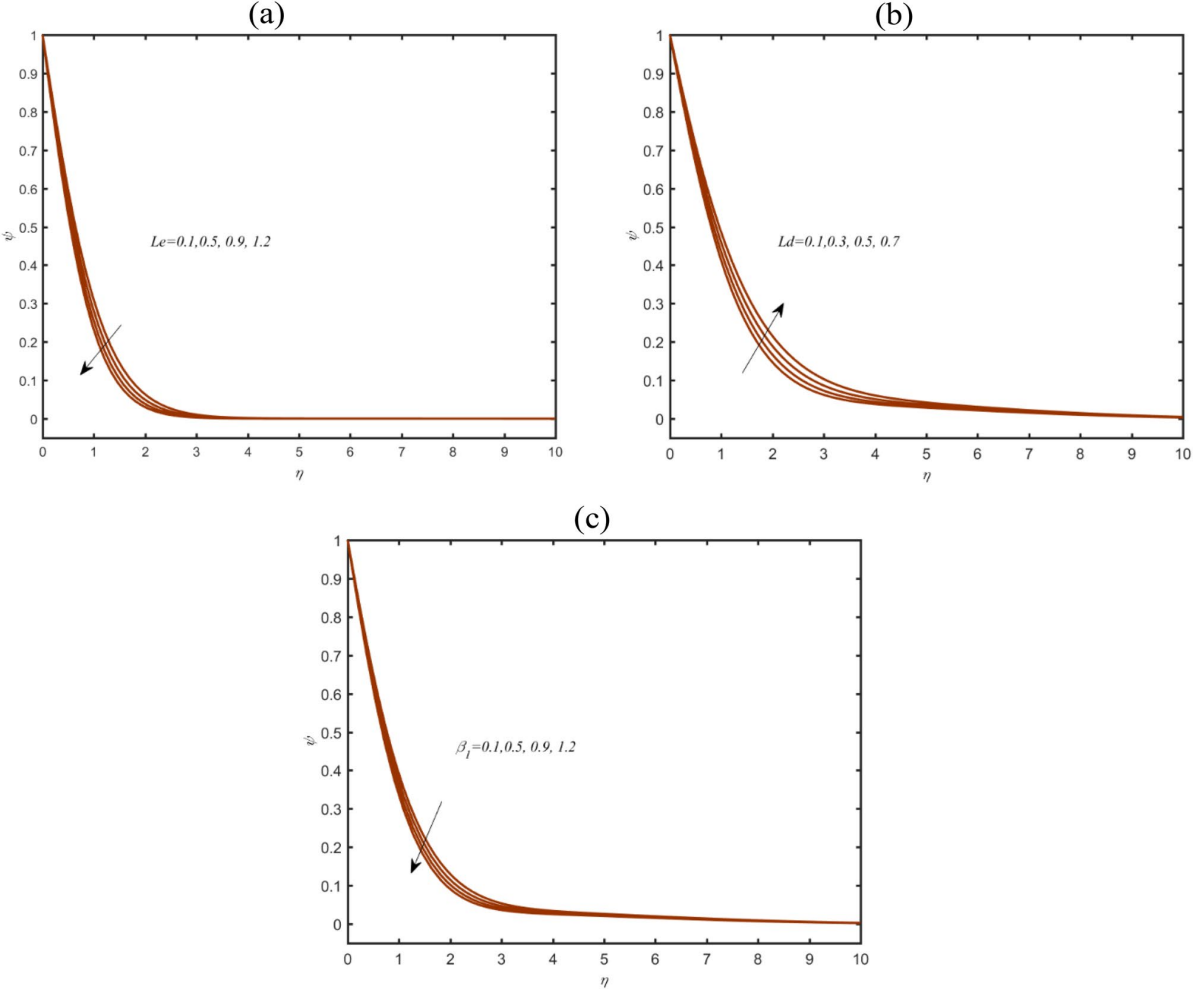
(**a**–**c**): Profile of $\psi$ due to (**a**) $Le$ (**b**) $Ld$ (**c**) $\beta_{1}$.

Figure 5a exclusively reports the dynamic of solutal concentration $\psi$ due to regular Lewis number $Le$. The find out observations convey that $\psi$ is lower with $Le$. The physical theme behind this declining trend is due to reverse relation between $Le$ and species diffusion. The deviation in $\psi$ Dufour Lewis number $Ld$ has been noticed in Fig. 5b. For peak values of $Ld$, the solutal concentration of nanoparticles enriches also. Physically, the association of $Ld$ with Lewis number comprising smaller mass diffusivity. Figure 5c depicted the outcomes for observations for thixotropic parameter $\beta_{1}$ on $\psi$. A decrement is judged out in $\psi$ due to $\beta_{1}$.

**Figure 6:**
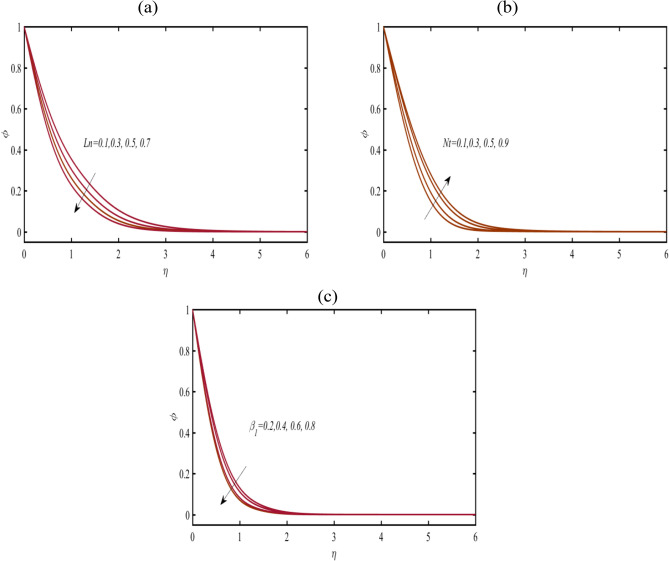
(**a**–**c**): Profile of $\phi$ due to (**a**) $Ln$ (**b**) $Nt$ (**c**) $\beta_{1}$.

Figure 6a pronounced the assessment in nanoparticles concentration $\phi$ due to nano-Lewis coefficient $Ln$. The declining variation $Ln$ on $\phi$ is objected. Physically, the nano-Lewis number is associated to ratios between thermal to mass diffusivities. The role of $Ln$ is important for natural convection phenomenon in heat and mass transfer framework. Figure 6b have been prepared to analyze the silent contribution of thermophoresis parameter $Nt$ on $\phi$. The nanoparticles concentration enhanced for $Nt$ due to thermos-diffusion phenomenon. A slight change in nanoparticles concentration is visualized due to migration of heated particles in cooler space. Figure 6c pronounced that $\phi$ declined when $\beta_{1}$ varies.

**Figure 7:**
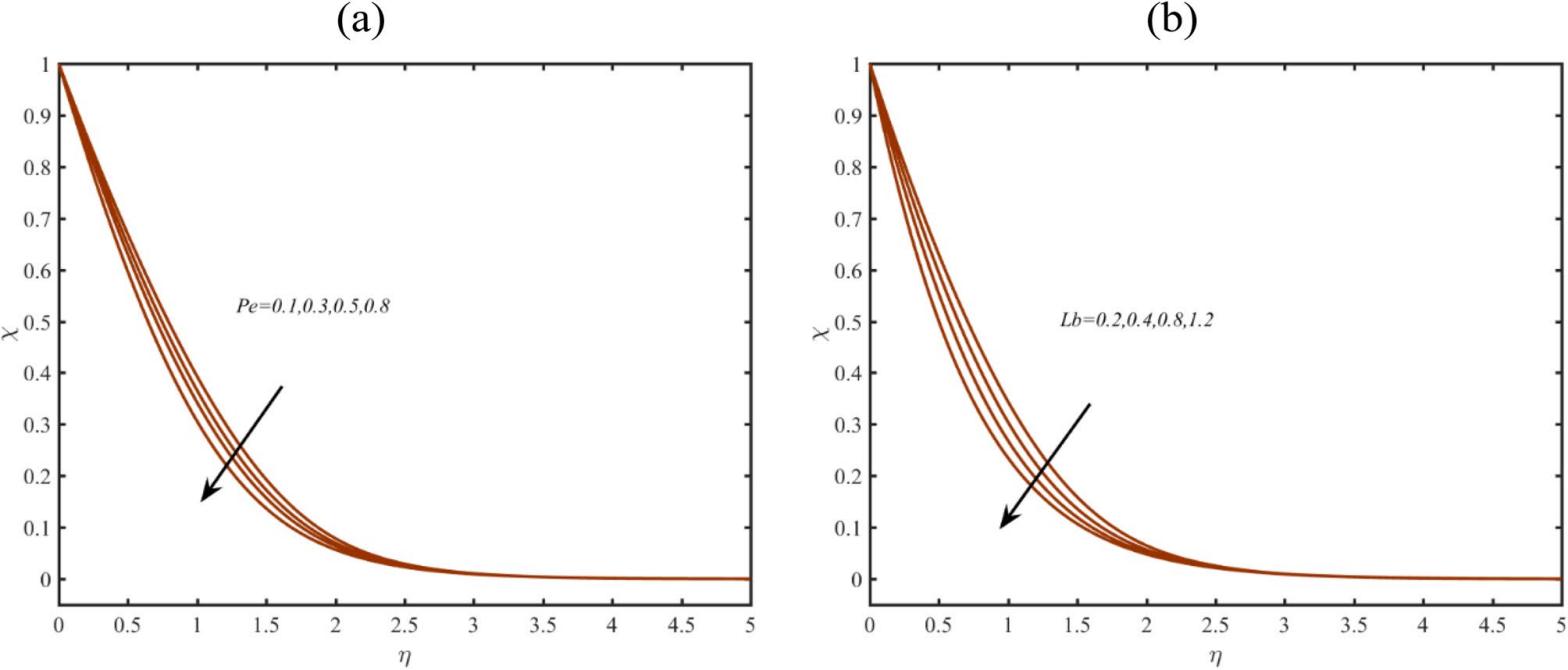
(**a**–**b**): Profile of $\chi$ due to (**a**) $Pe$ (**b**) $Lb$.

The analysis is performed in Fig. 7a,b for highlighting the change in microorganism profile $\chi$ for bioconvection Lewis number $Lb$ and Peclet number $Pe$. Lower deduction is visualized in profile of $\chi$ with changing $Lb$ and $Pe$. Physical aspects of this silent features due to $Pe$ are based on low motile diffusivity.

prepare the observations for $- \theta^{\prime}\left( 0 \right), - \varphi^{\prime}\left( 0 \right), - \phi^{\prime}\left( 0 \right)$ and $- \chi^{\prime}\left( 0 \right)$ when different parameters get varied. All physical quantities increases for magnetic parameter $M$ and thixotropic parameters $\beta_{1}$ and $\beta_{2} .$ Reverse observations are presented when $Nd$ enhances.

## Conclusions

This investigation has presented the significance of triple diffusion phenomenon associated to the thixotropic nanofluid. The mathematical model was further supported with applications of induced magnetic force. The numerical simulations are performed with shooting method. Major conclusions are:

- The enhancement in thixotropic parameter and velocity ratio constant leads to increment in velocity.
- The increasing effects of reciprocal Prandtl number and magnetic parameter on induced magnetic field profile has been exhibited.
- A declining assessment in induced magnetic field profile is exhibited due to thixotropic parameter.
- The heat transfer rate enhances due to Dufour number while declining outcomes are noted against thixotropic parameters.
- With magnetic parameter, the temperature rate can be controlled.
- The solutal concentration enhances for Dufour Lewis number.
- The nanoparticles concentration is lower due to thixotropic parameter and nano-Lewis number.
- The summarized results may present significance in solar energy, plasma physics, thermal processes, chemical systems, membrane transport, astrophysics, manufacturing processes, nuclear reactors etc.

## Data Availability

The data that support the findings of this study are available from the corresponding author upon reasonable request.
